# Measurement of Fluorescence in a Rhodamine-123 Doped Self-Assembled “Giant” Mesostructured Silica Sphere Using a Smartphone as Optical Hardware

**DOI:** 10.3390/s110707055

**Published:** 2011-07-06

**Authors:** John Canning, Angelica Lau, Masood Naqshbandi, Ingemar Petermann, Maxwell J. Crossley

**Affiliations:** 1 Interdisciplinary Photonic Laboratories (iPL), School of Chemistry, The University of Sydney, NSW 2006, Australia; E-Mails: lwyangelica@gmail.com (A.L.); mnaq6951@uni.sydney.edu.au (M.N.); ingemar.petermann@gmail.com (I.P.); 2 School of Chemistry, The University of Sydney, NSW 2006, Australia;E-Mail: maxwell.crossley@sydney.edu.au

**Keywords:** biological sensing and sensors, optical diagnostics for medicine, fluorescence, optoelectronics, light-emitting diodes, fluorescence microscopy, nanomaterials, silica, optical instruments, smartphones, mobile platforms

## Abstract

The blue OLED emission from a mobile phone was characterised, revealing a sharp emission band centred at *λ* = 445 nm with a 3dB bandwidth Δ*λ* ∼ 20 nm. It was used to excite Rhodamine 123 doped within a “giant” mesostructured silica sphere during fabrication through evaporative self-assembly of silica nanoparticles. Fluorescence was able to be detected using a standard optical microscope fitted with a green transmission pass filter and cooled CCD and with 1 ms exposure time demonstrating the potential of mobile platforms as the basis for portable diagnostics in the field.

## 1. Introduction

Mesostructured silica spheres generally are considered a suitable candidate for a range of chemical delivery applications including fluorophore marker transport and drug treatment when sufficiently small [1]. Both solid and hollow spheres can often be produced chemically through reaction after rapid evaporation of hydrolyzed silicon alkoxide—surfactant solutions [2,3], often in spray form. Here, we report on the fabrication of giant (1.5 mm) mesostructured spheres through slow solvent evaporation from the liquid phase of a solution of prepared silica nanoparticles. A super hydrophobic surface overcame surface inhibition to the shell formation. We also report a novel use of a mobile platform—in this case a Smartphone—as optical hardware to excite fluorescence, demonstrating a new approach to portable diagnostics that potentially overcomes many of the limits of existing methods. For example, hardware and data processing limitations are often used to justify the push to low cost lab-on-a-chip [4] technologies, and other compact, disposable optical based sensor technologies, in order to bring affordable instant diagnostics in remote and isolate regions. There are no other viable alternatives at present.

For biodiagnostics, fluorescence remains a key testing method in determining quickly the health status of a sample under test (whether it is from a human, a water ecosystem or so on). Fluorescence demands novel integration of both an excitation source and a detector. Two ways are adopted towards this approach: one is to make a handheld device into which a sample on a disposable slide or microfluidic chip is inserted [5]; the second is to integrate totally the excitation source and detector onto the disposable chip using mass cheap technologies such as those based on inkjet deposition [6].

There are several justifications for the approach proposed here: (1) smart mobile phones commonly used today have state-of-the-art active matrix organic light emitting diode (“AMOLED”) technology, introduced in 2011, driven by a large telecommunications market—as a consequence, this technology has received, and will continue to receive, significant investment ensuring the technology remains leading edge both in terms of the OLED on an amorphous (in some cases polycrystalline) silicon transistor backplane and in terms of the manner in which they can be programmed; (2) mobile platforms are increasingly being considered for signal processing and transmission of sensors which can potentially clip into the device through standard ports [7]; (3) mobile platforms are commonly available in remote regions and villages where, paradoxically, power may not be available for other more essential items. We therefore merge the ideas together and suggest that recent technological developments of mobile platforms (including tablet computers) are making them ideal as universal optical hardware equipment, extending previously proposed ideas for mobiles as software processers or control units for transmitting data or controlling equipment [7,8]. As proof-of-principle we consider the requirement of an excitation source and characterise a standard android platform. The open source platform means it is relatively easily for specific programs to be written to control Google developed Java libraries; in fact, there is already a simple application freely available to control the LED emission profile (by adjusting each OLED type) and intensity [9]. Although in this work we focus on OLED technology, it is clear other competing processes are now equally advanced, including recent liquid crystal display (LCD) technologies on an amorphous silicon backplane [10].

## 2. Smartphone Technology

**Figure 1.**
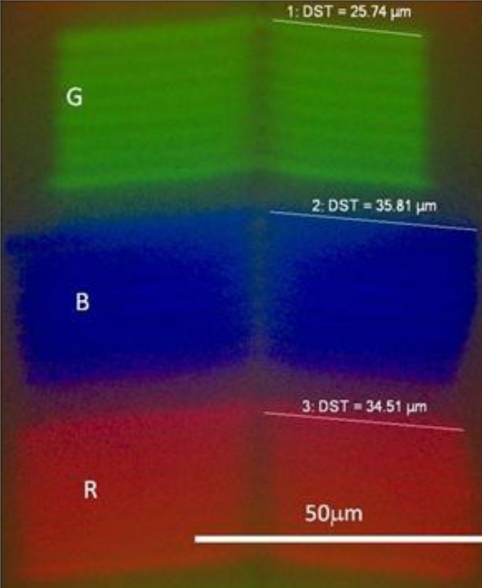
Microscope image of RGB OLED on android screen for Smartphone HTC Desire. Approximate dimensions of each pixel are shown.

**Figure 2.**
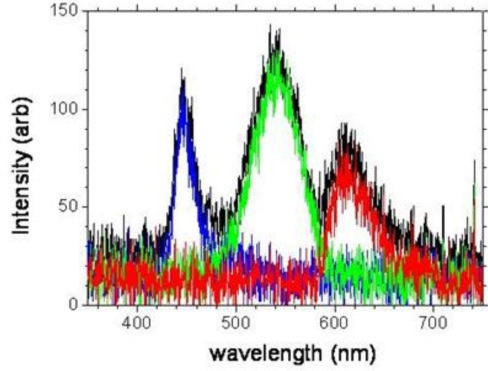
Optical spectra showing the emission from individual RGB OLED and the total spectra when all are activated.

The mobile platform used is the android HTC Desire readily available commercially and which has a relatively large screen area. We chose the android approach purely because it has open source code potentially allowing ready programming of the equipment for specific applications. Figure 1 shows the individual RGB unit OLED components when examined under an optical microscope and camera. Evidently, the length of each component appears adjusted to account for spectral emission variations and average human eye sensitivity variation across RGB. Hence, by individually addressing each OLED its possible to select only the blue, green or red. Some spectral shaping is possible by addressing any combination thereof of these. This ability to individually select each OLED, using available or custom software applications, is part of the inherent power of these Smartphones. When examined with a fitted spectrometer to the microscope, the spectral profiles, shown in Figure 2, are well defined and distinct showing very good RGB contrast, making them suitable as individual fluorescence excitation sources. The blue band is centred at 445 nm and has a 3 dB bandwidth of ∼20 nm, making it suitable for a number of fluorescent markers.

## 3. Silica Mesostructured Spheres

**Figure 3.**
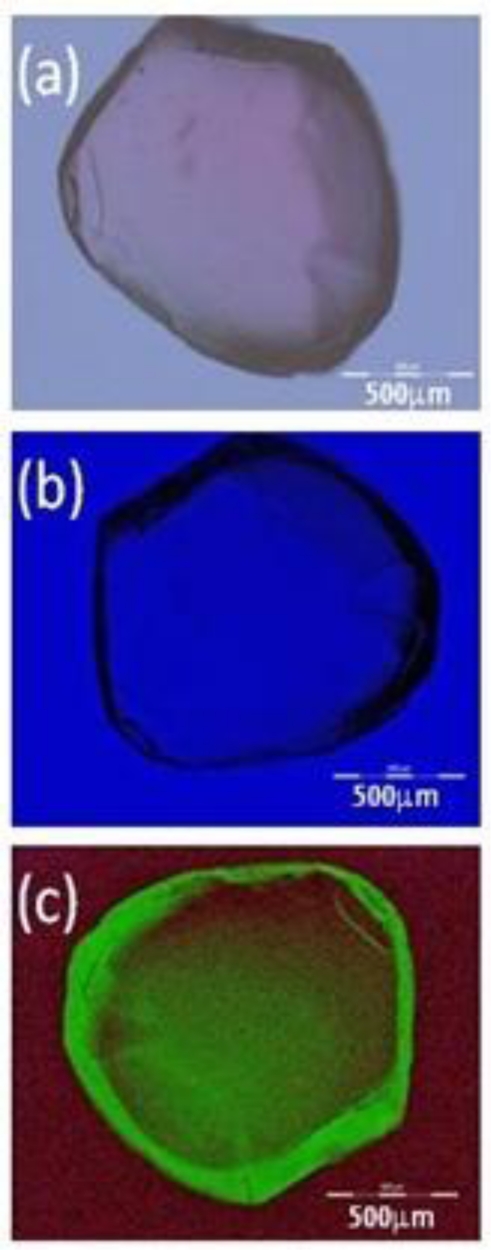
Images obtained using the Smartphone as optical source: **(a)** Optical image of hollow shell using all RGB components; **(b)** Optical image using just the blue component and **(c)** fluorescent image obtained by excitation with the blue and transmitting green only.

The sample to be tested is a silica mesostructure sphere shown in Figure 3(a). It was fabricated by evaporative self-assembly of a silica solution containing colloidal silica nanoparticles (40 wt% SiO_2_), measured by dynamic light scattering to have a size distribution of 20–30 nm [11], in water. A small amount of NH_4_^+^ prevents aggregation of the silica nanoparticles in solution. In contrast to surfactant based formation of silica mesostructures, where the evaporation rate was optimised by mixing water with ethanol [3], it was found that when starting with nanoparticles the slower the evaporation the larger the spheres and the less likely the cracking. Therefore, no ethanol is required and the evaporation is carried out more slowly with just water. Drops of 10 μL volume were deposited onto a super hydrophobic surface prepared by treating a silver-coated copper plate with heptadecfluoro-1-decanethiol (HFDT) [12]. Upon evaporation (295 K, 1 atm), aggregation and van der Waals attraction led to spheres being formed—contact angles were measured by a small CMOS camera to be *α* ∼ 150°. The structures tended to be increasingly distorted in shape with enormous potential energy arising from their formation—a few were observed to explode upon drying. The quality of the super hydrophobic surface was also observed to play an important part in the quality of sphere formed. Along with their distorted shape, many were sensitive to perturbations and readily split into halves. In order to investigate this more closely and the possibility of introducing dopants, the fabrication process was repeated by incorporating Rhodamine 123 laser dye, which has a peak absorption at 505 nm and a laser/fluorescence emission ∼560 nm in the green [13]. Generally it was observed that the organic dye appeared to stabilise the formation of the giant spheres allowing spheres up to 1.5 mm to be fabricated with less splitting than that obtained without the use of the dye. One of the larger, distorted spheres that did split into two halves during handling was used as the test sample.

## 4. Experimental Results

The optical source used to excite this structure on a combined optical and fluorescence microscope set-up was the android mobile Smartphone (HTC Desire). The sample was placed directly on the large area AMOLED screen. Using a freely downloaded application [9], in addition to the white light of all RGB bands together, each RGB component can be selected—specifically, the Rhodamine 123 fluorescence is excited using the 445 nm band.

The hemisphere is taken off the super hydrophobic surface and placed onto a glass slide which in turn is placed across the mobile phone screen. Figure 3(a) shows an optical image obtained with the mobile phone using all RGB components. It is approximately 1.5 mm in diameter. A slight reddish tinge is observed from within. Figure 3(b) shows the image when only blue light is emitted—there is no filtering of the light in the microscope. The clear blue optical transmission inside the sphere suggests it is highly uniformly packed with little scattering, in contrast to the edges. In Figure 3(c) a filter (>20 dB contrast) is used to cut out the blue and only allow fluorescing green through. A gain-charged, cooled CCD camera was used to detect the weak signal, where integration was performed over 1 ms. The shell thickness is estimated to be <20 μm.

From this experiment, Rhodamine 123 was successfully integrated into a “giant” silica mesostructure sphere; it appears to be mostly concentrated at the edge of the sphere where the packing density is less uniform and scattering is highest. Based on the number of spheres formed without cracking or exploding during fabrication, the presence of the organic dye near the surface of the spheres helped to stabilise the formation of these spheres, perhaps by reducing the condensation rate and allowing other material to escape. The total integration of dye in silica was ascertained to be low given the relatively long integration time required; although we also note that excitation at 445 nm was on the short wavelength side of the peak absorption at 505 nm. The concentration of dye within the sphere could not be determined and appears to vary between the centre of the sphere and the outer “shell”; regardless, a more suitable fluorescent marker will likely give stronger results.

**Figure 4.**
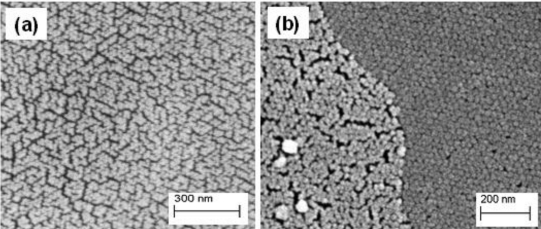
SEM images of a regular sphere surface and edge of fracture showing dense hexagonal close packing of the structure. A very thin surface layer shows marked porosity over the interior and may partially explain the observed green fluorescence intensity at the edges in Figure 3(c)

SEM imaging of other similar spheres shows hexagonal close packing of the nanoparticles. There is, however, a thin porous more “disordered” layer at the surface which may account for the intensified green fluorescence observed there (Figure 4). The interior structure is well ordered by contrast.

## 5. Conclusions

The successful excitation demonstrates the first time a mobile platform has been used as optical hardware raising the possibility of an entirely new approach to practical sensing and diagnostics, including telecommunications and other signal infrastructure test equipment. Two factors make this possible—the state-of-the-art OLEDs on a low temperature prepared silicon transistor backplane and the open access android platform enabling total software control of the platform and its components. The large area screen of the mobile makes it straight forward to simply place a standard low-cost slide, or microfluidic chip, containing the specimen under test. Further, although we were restricted in use to a good optical microscope and fluorescence imaging camera in these experiments, hardware advances will undoubtedly permit the next generation of mobiles to perform integrated detector functions. For example, the existing CCD camera on many phones is capable of being programmed as a detector, although in most cameras the phone is on the opposite side to the screen (two may be used). Importantly, this technology is accessible to all including those within remote developing regions where mobiles have become an essential tool.

It is anticipated that by using custom tailored programs the OLED signal intensity can be raised significantly, bearing in mind potential limits in lifetime performance—pulsed operation will help mitigate these issues. Presently, AMOLEDS have relatively low irradiance (∼10,000 cd/me^2^), but this is improving. Given that these are state-of-the-art easily addressed OLEDs (and LCDs), rapid modulations should in principle be straightforward to program, allowing fluorescence decay measurements to also be undertaken, further increasing potential specimen discrimination
